# Unravelling Mechanisms of Doxorubicin-Induced Toxicity in 3D Human Intestinal Organoids

**DOI:** 10.3390/ijms23031286

**Published:** 2022-01-24

**Authors:** Daniela Rodrigues, Luke Coyle, Barbara Füzi, Sofia Ferreira, Heeseung Jo, Bram Herpers, Seung-Wook Chung, Ciarán Fisher, Jos C. S. Kleinjans, Danyel Jennen, Theo M. de Kok

**Affiliations:** 1 Department of Toxicogenomics, GROW School for Oncology and Developmental Biology, Maastricht University, 6229 ER Maastricht, The Netherlands; j.kleinjans@maastrichtuniversity.nl (J.C.S.K.); danyel.jennen@maastrichtuniversity.nl (D.J.); t.dekok@maastrichtuniversity.nl (T.M.d.K.); 2 Boehringer Ingelheim International GmbH, Pharmaceuticals Inc., Ridgefield, CT 06877, USA; luke.coyle@boehringer-ingelheim.com (L.C.); seung.chung@boehringer-ingelheim.com (S.-W.C.); 3 Department of Pharmaceutical Sciences, University of Vienna, Althanstrasse 14, 1090 Vienna, Austria; barbara.fuezi@univie.ac.at; 4 Certara UK Limited, Simcyp Division, Sheffield S1 2BJ, UK; sofia.ferreira1@astrazeneca.com (S.F.); emily.jo@certara.com (H.J.); ciaran.p.fisher@gsk.com (C.F.); 5 Crown Bioscience Netherlands B.V., J.H. Oortweg 21, 2333 CH Leiden, The Netherlands; bram.herpers@crownbio.com

**Keywords:** doxorubicin, toxicity, human organoid models, molecular mechanisms, transcriptomics

## Abstract

Doxorubicin is widely used in the treatment of different cancers, and its side effects can be severe in many tissues, including the intestines. Symptoms such as diarrhoea and abdominal pain caused by intestinal inflammation lead to the interruption of chemotherapy. Nevertheless, the molecular mechanisms associated with doxorubicin intestinal toxicity have been poorly explored. This study aims to investigate such mechanisms by exposing 3D small intestine and colon organoids to doxorubicin and to evaluate transcriptomic responses in relation to viability and apoptosis as physiological endpoints. The in vitro concentrations and dosing regimens of doxorubicin were selected based on physiologically based pharmacokinetic model simulations of treatment regimens recommended for cancer patients. Cytotoxicity and cell morphology were evaluated as well as gene expression and biological pathways affected by doxorubicin. In both types of organoids, cell cycle, the p53 signalling pathway, and oxidative stress were the most affected pathways. However, significant differences between colon and SI organoids were evident, particularly in essential metabolic pathways. Short time-series expression miner was used to further explore temporal changes in gene profiles, which identified distinct tissue responses. Finally, in silico proteomics revealed important proteins involved in doxorubicin metabolism and cellular processes that were in line with the transcriptomic responses, including cell cycle and senescence, transport of molecules, and mitochondria impairment. This study provides new insight into doxorubicin-induced effects on the gene expression levels in the intestines. Currently, we are exploring the potential use of these data in establishing quantitative systems toxicology models for the prediction of drug-induced gastrointestinal toxicity.

## 1. Introduction

Doxorubicin (DOX) is a chemotherapeutical drug that belongs to the class of anthracyclines, being first isolated from *Streptomyces peucetius var. caesius* in 1967 [1]. DOX has application in the treatment of a wide range of cancers, such as solid tumours, acute myeloblastic and lymphoblastic leukaemia, breast, ovarian, prostate, gastric carcinomas, osteosarcomas, and soft tissue sarcomas [2]. Despite being one of the most standardized and recommended drugs to treat such malignancies in the last 40 years, DOX is often associated with severe side effects. Common side effects include hair loss, vomiting, weight loss, rash and suppression of the bone marrow. DOX is also associated with rather serious effects, including cardiotoxicity, leading to cardiomyopathy and subsequently congestive heart failure, hepatotoxicity, erythema, and disruption of the intestinal epithelium [2,3]. Disruption and subsequent inflammation of the intestinal epithelium induce symptoms such as diarrhoea, vomiting, abdominal pain and nausea. Consequently, cancer treatments can be compromised, dramatically impairing patient’s survival and quality of life.

The mechanisms by which DOX exerts its toxic effects in the intestinal cells are not fully understood yet. Although it is known that it targets and kills proliferative malignant cells, which divide at a higher rate than healthy ones, DOX is not cancer cell-specific and can, therefore, also affect healthy cells of multiple organs, leading to severe damaging effects even at therapeutic doses [3]. There are two proposed mechanisms of action linked to DOX: (1) intercalation into DNA and disruption of DNA topoisomerases and (2) generation of reactive oxygen species (ROS) [4,5]. The first mechanism leads to the unwinding of DNA, DNA replication, RNA transcription and translation, and ultimately, protein biosynthesis. Consequently, cell cycle is interrupted, and cells stop proliferating [4,6]. This mechanism is thought to be the main anti-cancer activity of DOX, whereas the generation of ROS is more associated with its toxic effect [7]. ROS can be generated as a result of DOX metabolism, in which a semiquinone, a rather unstable and oxidative molecule, is formed at complex I of the electron transport chain (ETC) [8]. Oxidative stress causes membrane damage, DNA damage, mitochondria dysfunction, lipid peroxidation, and trigger cell death pathways [3,4,8]. Taken all together, these mechanisms can be associated with DOX-induced toxicity due to inflammation, impairment of mitochondria and ATP synthesis, and ultimately the induction of apoptosis [3,9]. However, the molecular mechanisms through which DOX causes intestinal damage have not been investigated as the great majority of studies focus on cardiotoxicity. For this reason, this study aimed at not only confirming the hypothetical mode of action of the drug, but mainly at generating new data to advance our knowledge of the mechanisms involved in DOX-induced intestinal toxicity. To accomplish this, high-throughput transcriptomic analysis was performed on innovative 3D culture models of colon and small intestine (SI) organoids, derived from human tissue biopsies [10].

The development and application of three-dimensional (3D) culture systems in various fields, including disease modelling, drug discovery, screening, and drug target identification has exponentially increased as these models replicate tissue-like structures and characteristics more accurately than monolayer cultures [11,12]. Moreover, the introduction of extracellular matrices (ECM), e.g., matrigel [13], in the establishment of 3D cell cultures has enabled the replication of cell–environment interactions, leading to augmented cell proliferation, differentiation, and cellular functions [12]. This is important for creating cell culture conditions similar to the environment within tissues/organs, particularly in cancer and anticancer drug research. Several studies have been conducted on 3D culture technologies in which these are reported as potential tools to investigate drug combinations for the treatment of cancer, drug responses, and chemoresistance profiles [14,15,16]. More related to the gastrointestinal (GI) tract, the investigation of GI tissue development, homeostasis, diseases, and treatments has greatly benefited from 3D organoid models [17,18]. Similarly, intestinal organoids have shown to possess key features of human in vivo cells, i.e., they show similar cellular organization, behaviour, and crypt-like structures, which are more advantageous than other cell and rodent models [19,20]. Therefore, 3D cell culture technologies hold promise in overcoming the limited cell conditions and drug responses observed in 2D systems [11], and, consequently, in improving pre-clinical drug development studies.

In this study, we hypothesized that DOX could affect colon and SI tissues differently since they have different cell physiology, dynamics, and function. Therefore, distinct gene expression profiles would be observed reflecting distinctive response mechanisms in both organoid types. The exposure concentrations of DOX were based on predictions from physiologically based pharmacokinetic (PBPK) model simulations to better represent the clinical dose regimens during cancer therapy [20,21,22]. Cytotoxicity measurements were evaluated and checked if they were in line with the transcriptomic responses. In addition, proteomics data were assessed through computational simulations [23,24] based on known DOX protein targets in the gut available in online repositories [25,26,27,28]. These data were useful to assess if target proteins were reflected on the transcriptomic data generated on the organoids, as well as to gain a broader insight into the drug mechanisms of toxicity. The ultimate goal of this study is to further apply the new transcriptomic data and molecular gene markers in quantitative systems toxicology (QST) models to predict drug-induced GI toxicity (transQST project).

## 2. Results

### 2.1. PBPK Simulation for Selection of DOX In Vitro Concentrations

**Figure 1:**
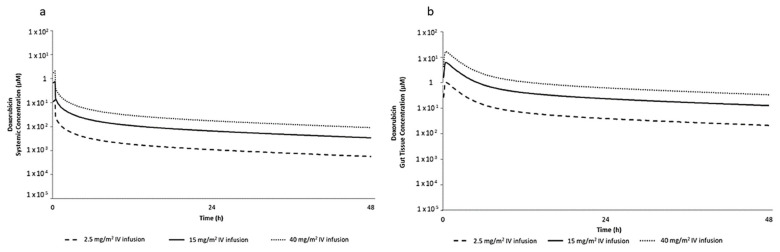
Predicted (**a**) mean total systemic plasma concentration and (**b**) mean total gut tissue concentration of DOX following 2.5, 15 and 40 mg/m^2^ intravenous (IV) dose infused over 20 min in humans.

The predictive performance of the DOX PBPK model was verified against clinically observed total DOX concentrations in plasma (Figure S1). Observed plasma concentrations of DOX were generally captured within the 95% confidence interval of the simulated plasma concentration-time profile. Figure 1 shows the predicted pharmacokinetic profiles of systemic and gut DOX concentrations following 20 min infusions of 2.5, 15, and 40 mg/m^2^ DOX, respectively, in humans. The gut tissue C_max_ was selected as the target exposure level for in vitro experiments as it provides relevant tissue exposure and a ‘worst-case scenario’ to inform a conservative safety assessment. PBPK predicted gut tissue total concentrations were eight-fold higher than that of total plasma concentration for all doses simulated.

Based on the PBPK predicted DOX C_max_ in gut tissue, the nominal concentration to achieve equivalent intracellular steady-state concentration in human intestinal organoid in vitro was predicted using the VIVD model. Table 1 shows the PBPK predicted gut tissue C_max_ for the three dosing regimens and the VIVD predicted in vitro nominal concentration. A ratio of 1.10 between total intracellular concentration and nominal test concentration was predicted, which is in line with DOX as a fairly lipophilic compound (Log P_ow_ of 1.27, [22]). These results were used to inform DOX dose ranges for subsequent in vitro experiments. The VIVD model assumes a monolayer cell culture, which is not representative of human intestinal organoids. However, the VIVD model does account for non-specific binding to medium components and plastic culture-ware and can still inform on the design of in vitro studies using organoids.

### 2.2. Cytotoxicity Evaluation of Colon and SI Organoids: Viability and Apoptosis after Exposure to DOX

**Figure 2:**
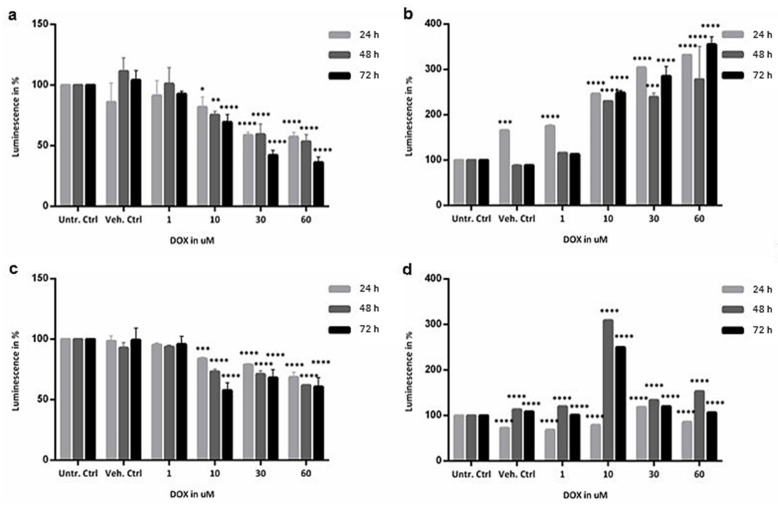
Functional assessment of healthy colon: (**a**) viability and (**b**) caspase 3/7 activation; and of SI organoids: (**c**) viability and (**d**) caspase 3/7 activation, when exposed to 1, 10, 30 and 60 µM DOX for 24 h in light grey, 48 h in dark grey and 72 h in black, compared with untreated controls. Values are in % of Luminescence. SD was calculated for each condition. Ctrl, control; DOX, doxorubicin; SD, standard deviation; SI, small intestine; Unt, untreated; Veh, vehicle. * *p* value of 0.03; ** *p* value of 0.002; *** *p* value of 0.0004; **** *p* value of 0.0001.

Assessment of viability of organoids was based on quantification of ATP levels, whereas apoptosis was assessed with caspase 3/7 activation assay. Overall, as shown in Figure 2, the lowest dose (1 µM) did not have any significant effect on cell viability and caspase 3/7 activation at all time points, in both colon and SI organoids. The temporal concentration effect of DOX exposure was more evident at 10, 30 and 60 µM. ATP levels tended to decrease across time and concentration in both organoids, whereas caspase 3/7 activation tended to increase in colon organoids but not as much in SI.

ATP levels of colon organoids treated with 10 µM of DOX decreased by 20 to 30% (*p* value = 0.0004). All time points were similar, and thus time did not have much effect at this concentration. Conversely, time played a more active role in the exposure of SI organoids, particularly at 72 h where ATP levels decreased by 40% (*p* value = 0.0001). Regarding the concentrations 30 and 60 µM, ATP levels decreased more significantly in the colon than in SI, particularly at 72 h. In colon organoids, differences between 24 and 48 h were not significant, but at 72 h, ATP levels decreased by 60% at 30 µM and more than 60% at 60 µM DOX, compared to the untreated controls (Figure 2a) (*p* value = 0.0001). In SI organoids, ATP levels decreased as drug concentrations increased. The effect of the exposure was similar in SI, where ATP decreased by 20% to 50% in all concentrations compared to the untreated controls (Figure 2c) (*p* value = 0.0001). Therefore, considering only ATP levels, colon and SI organoids were similarly affected by DOX.

Conversely, caspase 3/7 activation in colon organoids was more affected by the drug concentration with over three-fold difference between the untreated and treated groups (Figure 2b) (*p* value = 0.0001). Similarly, in SI organoids, caspase 3/7 activation was also significantly affected by the drug concentration compared to the untreated controls (*p* value = 0.0001) (Figure 2d). Moreover, there was a three-time increase in caspase 3/7 activation at 48 and 72 h when exposed to 10 µM, for which there is no clear indication of whether these are outliers or biological responses since transcriptomic analysis did not show significant changes in apoptosis or cell cycle-related genes for these particular treatment conditions in SI. In summary, although caspase 3/7 activation was significantly affected in both organoids and mainly concentration-dependent rather than time-dependent, it seems that in colon organoids the impact of the exposure to DOX was higher and more progressive across treatment conditions.

### 2.3. Image Analysis

**Figure 3:**
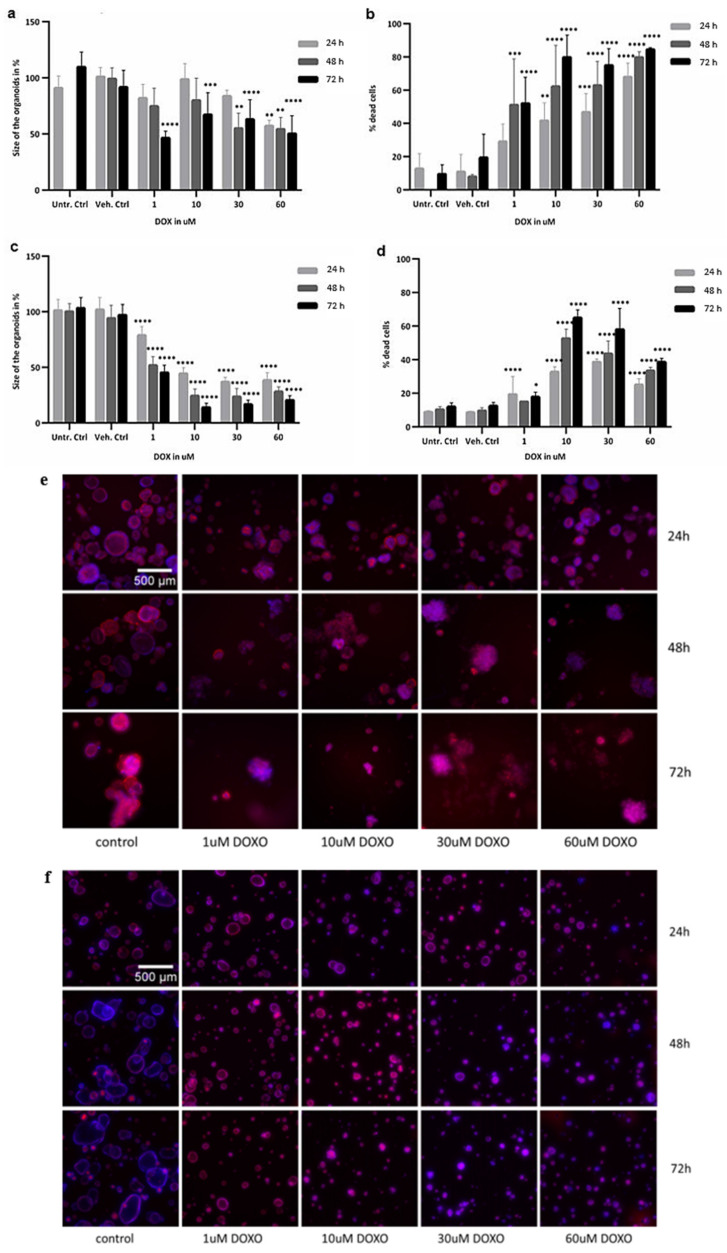
Morphological changes assessed through imaging analysis of healthy colon: (**a**) size, (**b**) percentage of cell death, and (**e**) microscope image analysis; and SI organoids: (**c**) size, (**d**) percentage of cell death, and (**f**) microscope image analysis, when exposed to 1, 10, 30 and 60 µM DOX for 24 h in light grey, 48 h in dark grey and 72 h in black, compared with untreated controls. Values are in % based on fluorescent intensity for each measured parameter. SD was calculated for each condition. Ctrl, control; DOX, doxorubicin; SD, standard deviation; SI, small intestine; Unt, untreated; Veh, vehicle. * *p* value of 0.04; ** *p* value of 0.008; *** *p* value of 0.0009; **** *p* value of 0.0001. Staining in control wells: Phalloidin-FITC (actin, in red) and Hoechst (DAPI channel, nuclei, in blue); treated wells: DOX bound to the nuclei (TRITC channel, nuclei, in blue); 1 pixel is 3.25 µm.

In addition to the evaluation of cell viability and caspase 3/7 activation, morphological changes caused by DOX, including size and volume of the organoids, and percentage of cell death, were observed after image processing (Figure 3). Total and average size of the colon organoids (Figure 3a) did not significantly decrease after 24 h exposure to DOX, whereas at higher concentrations after 48 and 72 h, the organoids became significantly smaller (*p* value = 0.0001). Statistically significant changes in cell death were evident in all treatment conditions (*p* value = 0.0001), except for 1 µM at 24 h, with an increase by 80% for the highest concentration at 72 h (Figure 3b). Additionally, the total and average sizes of SI organoids were affected at 1 µM after 24 h treatment (*p* value = 0.0001) (Figure 3c). Cell death (Figure 3d) increased significantly in the SI organoids as well, particularly after exposure to 10 µM DOX (*p* value = 0.0001), with an increase up to 60%, lower than in colon organoids. Figure 3e,f show additional microscope images of colon and SI organoids, comparing the controls with the doxorubicin treatments. The auto-fluorescence of DOX was taken into account when comparing the differences in staining intensities such that the image analysis was independent of DOX staining interferences. The morphology image data confirmed the results of the viability and caspase 3/7 assays, demonstrating that DOX inhibited cell growth and activated cell death processes.

### 2.4. Identification of Biological Pathways and Gene Responses Affected by DOX

Gene expression data from DOX-exposed organoids were used to identify biological pathways and differentially expressed genes (DEGs) involved in intestinal toxicity induced by the drug. First, alignment of the reads to the whole human genome was performed, ranging between 65% and 81% in colon samples and between 57% and 68% in SI samples, and thus, further analysis could proceed. Second, two SI samples presented low-quality RNA (RIN < 7) and two SI samples yielded less than 5 million reads, the recommended cut-off for differential gene expression (DGE) analysis [29,30]. Thus, these samples were regarded as unsuitable for analysis. As a consequence, the measurements of 60 µM at 72 h in the SI were not included. All samples derived from colon organoids presented more than 5 million read counts. After Bonferroni correction, and considering adjusted *p* value < 0.05, a consistent concentration-related increase in the number of DEGs was found in both organoids. Across time of exposure, the number of DEGs was lower at 48 h and higher again at 72 h.

PCA score scatter plots were generated to further explore the gene expression differences between treated and untreated organoids and how the DOX concentration and treatment duration would affect these (Figure S2). Regarding colon organoids (Figure S2a), there was a clear separation between controls and treated samples, as well as between the different DOX concentrations, although the two higher concentrations clustered together on the right. In turn, PCA plot of SI organoids (Figure S2b) showed that the controls were also clustering together along with the lower concentration of DOX. Similar to the colon organoids, the two higher concentrations also appeared in the same cluster on the right. Therefore, when comparing the distribution of samples from the colon with SI organoids, in the colon, there was a more evident separation of samples and effect of concentration (PC1) and time (PC2). Conversely, in SI, both the effect of concentration and time (particularly from 24 and 48 to 72 h) could be observed in PC1, whereas in PC2, there seemed to be a slight influence of earlier time points (24 to 48 h). Overall, the concentration of DOX affected more the organoids than the duration of exposure as the variance was lower for the latter. This is in line with the fact that, due to the limited metabolic clearance in the organoid assays, the exposure concentration is constant, and thus, it is the main driver of the observed drug effects rather than the duration of exposure.

The DEGs obtained for each treatment condition were used to perform ORA using CPDB. As a result, an overview of the altered pathways for treated samples compared to vehicle controls was obtained, from which the most significantly overrepresented pathways were identified using the q values and the number of DEGs involved.

#### 2.4.1. Pathway Analysis across Time and Concentration in Colon and SI Organoids

Pathway analysis showed several biological pathways being affected by the drug. In both colon and SI, the most affected pathways were mostly related to cell cycle, the p53 signalling, and DNA methylation. Other pathways affected across the treatment conditions were metabolism (especially of lipids, amino acids, and carbohydrates), cellular senescence, oxidative stress-induced senescence, and DNA repair. Conversely, ATP synthesis was only evident at the higher concentration in the colon, whereas apoptosis was observed neither in the colon nor in SI organoids, except at 24 h, 10 µM. An overview of the q values of these pathways, across time and concentration, for both organoids, is displayed in Table 2. Moreover, from the q values of each of the selected pathways and the number of DEGs affected by DOX, the responses from the colon organoids were statistically more significant, and more pathways were significantly affected than in SI organoids.

Further analyses on how these pathways were perturbed in colon and SI organoids across time and concentration of the drug showed significant differences between both organoids’ responses, suggesting activation of different mechanisms in both cells. This is particularly the case for cell cycle and DNA repair mechanisms, which were highly affected at 24 h in the colon, with a tendency to become less affected across concentration and time, whereas in SI these pathways were only significantly affected at 10 µM, after 24 and 48 h exposure. Furthermore, in the colon, the p53 signalling pathway was highly affected at 24 h, especially at 10 µM, at which the peak of dysregulation of this pathway seemed to be reached, as across 48 and 72 h q values tended to increase. In SI, the p53 signalling pathway was similarly affected, with the exception that q values were not significant at 48 and 72 h. Metabolic pathways were only significantly affected in the colon: (1) glycolysis after 48 and 72 h exposure, with q values more significant across concentration; (2) respiratory electron transport and ATP synthesis became affected only at the highest concentration, particularly at 24 h; and (3) metabolism of lipids was more significantly affected at 48 h. Metabolism of amino acids was also different in the colon and SI. Whereas in SI, it was significantly affected at 24 and 48 h, in the colon, only became affected after 72 h exposure. Conversely, DNA methylation was significantly affected in both tissues, although q values were lower in the colon overall. Similarly, oxidative stress-induced senescence was highly affected in both organoids, becoming less affected across time and concentration. Although we measured increased caspase activities, at the gene expression level, apoptosis was not significantly activated throughout the treatment conditions, except for SI after 24 h exposure to 10 µM of DOX.

#### 2.4.2. Expression Profiles of DEGs Affected in Colon and SI Organoids

Following pathway analysis, the expression levels of DEGs were further investigated to check for trends of alterations in the expression profiles, focusing on genes involved in cell cycle, the p53 signalling pathway, respiratory electron transport and ATP synthesis, DNA methylation, and oxidative stress-induced senescence. Venn diagrams were used to identify DEGs that were in common and affected in the same direction of expression in the colon and SI (Figure S3), considering all time points and DOX concentrations. DEGs involved in respiratory electron transport and ATP synthesis were not found for SI organoids as this pathway was not significantly affected (Table 2).

**Figure 4:**
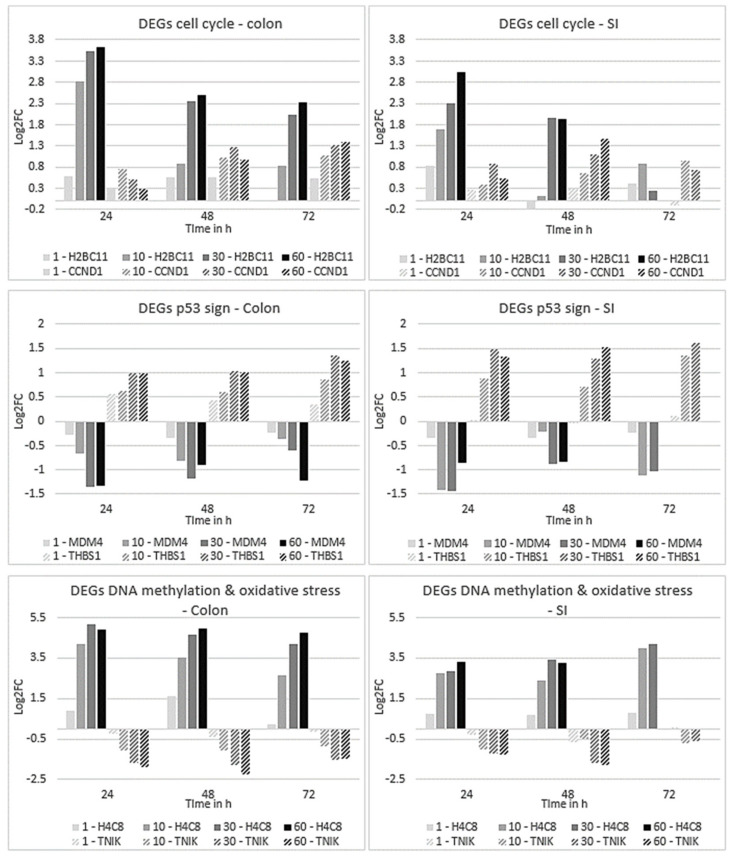
Gene plots representing the expression profiles of genes involved in cell cycle, the p53 signalling pathway, DNA methylation, and oxidative stress-induced senescence, after 24, 48 and 72 h exposure to all DOX concentrations of organoids derived from colon (on the left) and SI (on the right). Values for gene profiles are based on the log2FC. Plot colours correspond to the different concentrations of DOX: lighter grey represents 1 µM; grey represents 10 µM; dark grey represents 30 µM; black represents 60 µM; and DEG: with or without stripes.

Regarding cell cycle, expression levels of 41 genes were altered by DOX in both organoids. Most DEGs were histone encoding genes, apart from cyclins and kinases, p53, and MDM4 regulator of p53. The expression level changes across treatment conditions of the two top genes involved in cell cycle in common between the organoids, *H2BC11* (H2B clustered histone 11) and *CCND1* (cyclin D1), are sown in Figure 4. Regarding the gene *H2BC11*, expression levels in colon organoids decreased over time considering the same concentration, but for each time point, expression levels were increased until the highest concentration was reached. This trend of alteration was similar in all time points. Similarly, in SI organoids, the same pattern of alteration was observed, except at 72 h, as at 10 µM DOX, expression levels of *H2BC11* were higher as compared to the expression levels for the other concentrations. Moreover, expression levels of this gene were higher in the colon than in SI organoids. In turn, expression levels of *CCND1* tended to increase over time and concentration, particularly at 72 h in the colon and earlier at 48 h in SI.

As for the DEGs involved in the p53 signalling pathway, DNA methylation, and oxidative stress-induced senescence, 7, 16 and 23 genes were in common, respectively, between the organoids. Genes from each of these pathways were also selected to show the trend of alterations in their expression levels across all exposure conditions (Figure 4). Regarding the p53 signalling pathway, *MDM4*, a regulator of p53 activity, and *THBS1*, which encodes for Trombospondin-1, an endogenous inhibitor of angiogenesis and whose promoter is activated by p53 [31], were the top two common genes. These genes presented different alterations in their expression levels. The first one, *MDM4*, was downregulated across concentrations, whereas over time there was an increase in the expression levels at 48 h and downregulation again at 72 h. The second one, *THBS1*, presented a gradual upregulation trend across time and concentration, particularly at 72 h, and its expression levels were higher in SI than in the colon.

The gene *H4C8*, a different histone encoding gene involved in DNA methylation, had a similar change in the expression levels as the gene *H2BC11* in the colon, as it was upregulated across concentration; however, downregulated over time. In SI, *H4C8* was also upregulated across concentration, but over time, unlike in the colon, the trend was of upregulation as well. Lastly, the gene *TNIK*, involved in oxidative stress-induced senescence, which encodes for a protein kinase involved in activation of the WNT signalling pathway, was downregulated across time and concentration, except at 72 h, in both organoids.

Taken all together, cell cycle- and DNA methylation-related genes had the most significant changes in their expression levels, in which histone encoding genes were downregulated over time but upregulated across concentration, whereas cyclin D1 was upregulated over time and concentration, except at 24 h. Genes associated with the p53 signalling regulation *MDM4* was downregulated and angiogenesis inhibitor *THBS1* was upregulated, particularly across concentration as time did not have a significant impact. Oxidative stress-related gene *TNIK* was downregulated across concentrations and similar for each time point, affecting also the WNT signalling. Therefore, the trend of alterations of the DEGs expression levels involved in conservative pathways was similar in both organoid types. Despite the similarities, major differences in DEGs expression levels and specific pathways were observed between colon and SI. These are described in the section below after performing STEM analysis.

### 2.5. Time-Dependent Gene Clustering Analysis

The DEGs, found significantly affected by DOX, were also used in time-series correlation analysis, using the STEM tool, in which all time points and DOX concentrations were included. Most conditions presented more than one significant time-dependent gene cluster (*p* < 0.05), except the condition 1 µM DOX, which had one significant cluster for colon organoids and no significant cluster for SI (Figure S4), as the expression levels of DEGs was not significant enough to provide a relevant cluster. In the colon, the expression levels of DEGs among clusters were variable between concentrations, as gene expression levels seem to either decrease or increase, but mostly the latter. Regarding SI organoids, similar trends were observed, with time-dependent clusters showing either gene upregulation or downregulation. However, unlike in the colon, the most significant cluster for each condition showed a decrease in the gene expression levels over time.

An enrichment network analysis using NetworkAnalyst tool was performed with the genes listed in the most significant time-dependent cluster of each condition. For each condition, clusters with the same colour had the same expression profiles, thus they were considered as one group. Furthermore, time-dependent clusters with *p* value > 0.01 showed pathways not related to DOX-induced toxicity and with intestinal cells function, hence they were not further investigated. In colon organoids, at 1 µM, the most enriched pathways were cell cycle and the p53 signalling pathway. At 10 µM, pathways prevailing in clusters 1 and 3 were DNA methylation, replication, cell cycle-related processes, cellular senescence, the p53 signalling, and RNA expression. Cluster 2 showed enriched pathways related to the p53 signalling pathway and metabolism, including biosynthesis of steroids and amino acids, and carbon metabolism, which includes pyruvate metabolism and glycolysis. For the two higher doses (30 and 60 µM), the p53 signalling pathway remained the most relevant pathway along with, particularly, beta-alanine and histidine metabolism. Regarding SI organoids, it was overall observed that the number of enriched pathways was lower than in colon organoids. Moreover, most of the pathways found in SI were different from the ones found in colon organoids. At 10 µM, the most enriched pathways were pyrimidine metabolism and one carbon pool by folate. At 30 µM, pyrimidine metabolism remained as a relevant pathway followed by other metabolic pathways, particularly vitamin B6 and sphingolipid metabolism. For the highest concentration, the most relevant pathways were quite different, as they included HIF-1 and AMPK signalling pathways, RNA degradation, and glycolysis.

Following enrichment pathways analysis, the most significantly affected genes derived from those time-dependent clusters were investigated. As expected, the great majority of DEGs observed in the colon and SI were different. For the two higher concentrations of DOX (30 and 60 µM), the five most affected DEGs in the colon or SI were selected for further analysis and they are described in Table 3. These results demonstrate the tissue-specific responses that distinguish colon from SI organoids.

### 2.6. Proteome Analysis

DOX is a well-studied drug with many activity data points available in public repositories. The workflow that was used to obtain single protein targets of DOX generated a diverse rather diverse profile of 39 proteins connected to DOX. Besides the obvious mode of action target DNA topoisomerase 2-alpha, other enzymes such as carbonyl reductase 1, nitric oxide synthase (endothelial and brain), as well as different CYP enzymes are part of the list. Furthermore, transporters, such as the multidrug resistance-associated protein 1, the bile salt export pump, solute carriers, as well as the carrier albumin are present. After applying the gut tissue filter, the number of target proteins decreased to 19 UniProt entries (Table 4).

The NADH dehydrogenase iron–sulphur proteins are subunits of the mitochondrial membrane respiratory chain NADH dehydrogenase (Complex I). Disturbance of Complex I can lead to mitochondrial toxicity [32]. Pathological changes in the GI tract were linked to nitric oxide synthetase [33]. CBR3 variants were discussed concerning DOX disposition and toxicity [34]. Consequently, the target profile correlates with the toxic properties of DOX.

**Figure 5:**
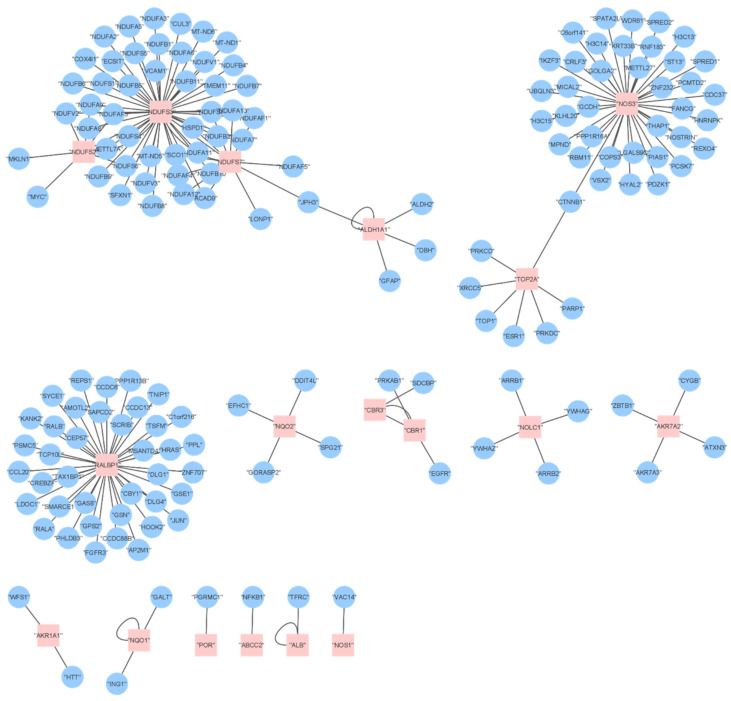
Network of direct targets (red, from Table 4) and their first-degree interactors (blue) affected by DOX. The topology of the network implicates a systemic effect of the drug.

The list of various targets predicts a diverse systemic effect of the drug. This hypothesis is supported by the interactome profile. The 19 tissue-specific targets directly interact with 164 proteins. The network is sparse with three highly connected hubs (Figure 5). There are several nodes with only one edge. The network topology implicates the systemic mechanisms triggered by DOX.

### 2.7. Comparing DOX Effects on Transcriptomics and Proteomics

**Figure 6:**
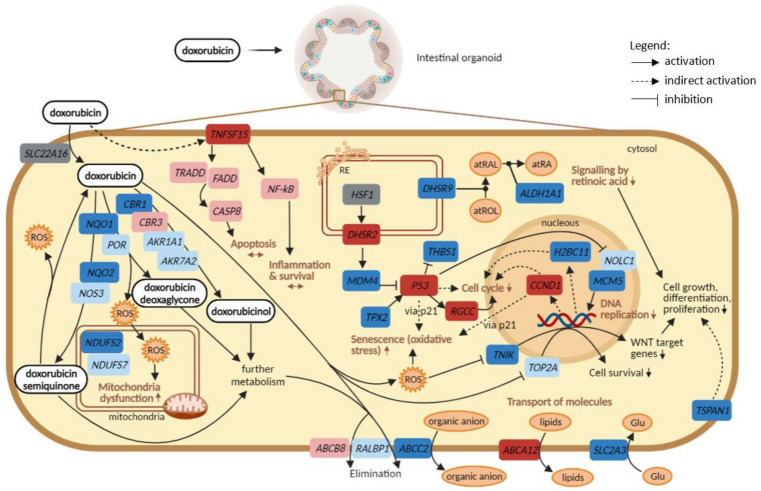
Comparison between transcriptomics and proteomics data, starting from DOX entrance to the cell to the several biological pathways and DEGs that are perturbed. For the alterations in the gene expression levels, concentrations of 30 and 60 µM were considered, at every time point. Genes in dark blue, significantly downregulated; light blue, not significantly downregulated; dark red, significantly upregulated; light red, not significantly upregulated; grey, not available. Image created with BioRender.com (accessed on 23 April 2021).

A comparison between the transcriptomic and proteomic findings was performed in which the most relevant DEGs and proteins affected by DOX in both organoids were considered, after exposure to the higher concentrations. A representation of the overall changes in the expression levels of genes and their consequences can be observed in Figure 6.

The proteins were considered as their encoding genes, and these include mostly proteins that are involved in the metabolism of DOX and its elimination (membrane transporters). Two of them though are involved in DNA replication, namely *TOP2A* and *NOLC1*. The proteomic data were subsequently compared to the transcriptomic data to check which of those proteins were encoded by significantly affected DEGs. Overall, 16 out of the 19 gene encoding proteins were found in both organoids, considering the higher concentrations of exposure (30 and 60 µM) at 72 h. Nevertheless, only gene expression levels of *ALDH1A1*, *CBR1*, *NQO1*, *NQO2*, *NDUFS2*, and *ABCC2* were significantly affected, particularly in the colon organoids as in SI organoids, only *ABCC2* was found significant.

In Figure 6, it is demonstrated that genes involved in DOX metabolism were downregulated after the exposure, except *CBR3*, which was upregulated, but not significantly. This is in line with the pathway analysis, as biological processes associated with drug metabolism were not found significantly affected. This could be linked to the fact that DOX metabolism is not the main function of intestinal cells [35] and, therefore, it does not prevail in the intestinal organoids, increasing the probability of intestinal damage caused by DOX and its metabolites. Similarly, genes that encode membrane transporters responsible for the elimination of DOX and its metabolites were downregulated. Consequently, DOX inside the organoid cells may lead to the generation of ROS, which in turn triggers several negative effects. One of them is the downregulation of *NDUFS2* and *NDUFS7*, both part of complex I of the respiratory electron chain, and consequent mitochondria dysfunction. Another consequence is upregulation of p53, in line with the observed relevance of the p53 signalling pathway, which correlates with changes in the expression levels of genes involved in cell cycle, DNA replication, and oxidative stress-induced senescence. In addition, ROS may lead to downregulation of *TNIK*, which contributes to the decrease in the transcription of WNT target genes.

Moreover, the exposure to DOX also caused downregulation of *DHSR9* (only in SI) and *ALDH1A1* (only in the colon), affecting signalling by the retinoic acid pathway. Next, other membrane transporters seem to be significantly affected, particularly *ABCA12* (upregulated only in the colon) and *SLC2A3* (downregulated only in SI), whose main functions are to export lipids and import glucose, respectively. Lastly, exposure to DOX led to the upregulation of *TNFSF15* in colon organoids. However, pathways activated by that gene, including apoptosis or inflammatory responses, were not significantly upregulated.

In summary, the results showed that genes involved in DOX metabolism were not significantly affected in contrast to those involved in its elimination, since genes encoding membrane transporters were downregulated. The consequent accumulation of DOX and its metabolites may lead to mitochondria complex I dysfunction, upregulation of the p53 signalling pathway, downregulation of WNT target genes and retinoic acid pathway. Additionally, transport of lipids and glucose were affected in the colon and SI, respectively. Apoptosis activation did not seem to be significantly modulated in either colon or SI.

## 3. Discussion

The main goal of this study was to investigate molecular mechanisms of toxicity of DOX in 3D human organoid models of both colon and small intestine. These new and promising cell culture models have shown to be suitable for the investigation of diseases, targeted therapies, drug development and screening overcoming the limitations of the 2D systems [11,12]. The ability to mimic the in vivo cell interactions, structures, and environment makes the 3D organoid models an advantageous alternative in research and pharmaceutical industry.

The 3D human colon and SI organoids were used to explore intestinal cell responses to DOX exposure on the gene expression level. Exposure concentrations were based on prediction from PBPK models used to simulate the clinical dosing that is usually recommended for cancer patient treatment. Organoids’ gene expression and in silico proteome responses were analysed to evaluate current hypotheses about DOX and to gain novel insights into the molecular mechanisms of action involved in toxic effects on intestinal epithelial cells at clinically relevant doses.

Overall, the responses observed in the colon were different from the responses observed in SI, despite some similarities. Cytotoxicity assays showed a similar trend in both organoids, although the decrease in ATP levels was stronger across conditions in colon than in SI organoids. Similarly, caspase activation assays showed similar increasing trends in both organoids, but it was more evident and progressive across treatment conditions in colon organoids. In SI, changes in caspase activation were not as strong as in the colon. Nevertheless, apoptosis or caspase-related pathways were not significantly affected in the organoids. Additionally, substantial morphological changes were also observed in both organoids with regard to the organoids’ size and percentage of cell death, demonstrating the toxic effects of the drug. Taken together, exposure to DOX led to a strong proliferation inhibiting effect in both types of organoids.

At the level of gene expression changes, exposure to DOX-induced alterations in cell cycle, DNA repair, and the p53 signalling pathway, which are closely related to the inhibition of DNA replication and RNA transcription caused by DOX [4,6] and thus in line with the hypothesis of DOX-induced mode of action. Moreover, alterations in the p53 signalling pathway influence gene responses involved in cell cycle arrest, ATP production, apoptosis and recruitment of inflammatory components (e.g., cytokines) [36,37]. These drug-induced alterations were more evident and consistent over time and concentrations in the colon than in SI. This could indicate that colon was not only more responsive towards DOX exposure but also that a distinct timing occurs in the gene expression between both tissues. The gene expression changes in the DNA methylation pathway were similarly affected in both organoids in a time and concentration-dependent manner. DNA methylation is an essential mechanism for normal cell development and for controlling gene expression [38]. Therefore, a possible hypothesis is that gene expression changes affecting DNA methylation could be an important mechanism through which DOX interferes with DNA/RNA related processes in the intestinal cells. Furthermore, oxidative stress and cellular senescence were found significantly affected by DOX in both organoids, in a time and concentration-dependent manner. These biological pathways can be related to the generation of ROS, thus supporting the hypothesis of the second mode of action associated with the drug [4,5]. Since oxidative stress caused by ROS leads to DNA damage, mitochondria impairment and lipid peroxidation [3,4,8], this mechanism can also be a major cause of the alterations in the biological pathways described above.

Novel findings indicated distinctive tissue responses in glycolysis and lipids metabolism, as they were only significantly affected in the colon. Both pathways are important in energy generation that fuels biological processes, including TCA cycle or fatty acid β-oxidation [39], and consequently, the normal function of mitochondria. By perturbing these pathways and impairing mitochondria functions, longer exposures to DOX can potentially lead to cell death, especially if the time for recovery is limited. Furthermore, and despite being relevant in both organoids, metabolism of amino acids was statistically significantly affected at earlier time points in SI whereas in the colon, only at 72 h. Since colon organoids seem to rely on other metabolic pathways that were not modulated in SI, namely glycolysis, respiratory electron chain, and lipids metabolism, this might explain the later changes in the metabolism of amino acids in the colon.

Time series analysis of gene expression changes showed additional differences between colon and SI organoid responses, as well as it provided new insights on the molecular changes induced by DOX. In colon organoids, cell cycle and the p53 signalling pathways were confirmed as the most relevant pathways along with energy generation metabolic pathways, including glycolysis, metabolism of pyruvate and amino acids, particularly metabolism of β-alanine and histidine, which showed to be relevant at the higher doses. Previously, it has been suggested that these two essential amino acids, apart from their role in energy production, might have antioxidant properties by participating in the scavenging of ROS and nitrogen species [40,41]. Therefore, metabolism of β-alanine and histidine are new findings in the colon, and they may be involved in the protection against oxidative stress caused by the drug in the colon cells. Regarding SI organoids, major effects were found in one-carbon metabolism mediated by folate, metabolism of pyrimidine, vitamin B6 and sphingolipids, HIF-1 and AMPK signalling pathways, and RNA degradation. One carbon metabolism mediated by the folate (vitamin B9) cofactor is essential for the maintenance of several biological processes including biosynthesis of nucleotides and redox defence [42]. Perturbations in the metabolism of pyrimidine, vitamin B6, important in the metabolism of amino acids [43], and consequent RNA degradation, are connected to one-carbon metabolism. As for sphingolipids metabolism, it was a unique and new finding in SI organoids. Sphingolipids are known key structural components of cell membranes, but they seem to be also involved in signalling pathways that regulate cell growth, differentiation, senescence, and apoptosis [44]. Similarly, HIF-1 and AMPK signalling pathways were also uniquely observed in SI organoids for the highest concentration (60 µM), being both involved in cell homeostasis and cellular adaptations to hypoxia [45]. A hypothesis could be that these new pathways resulting from SI cell responses to DOX are linked to the drop in cell viability (ATP levels), which didn’t necessarily reflect an increase in caspase activity. Additionally, the shift in carbon metabolism caused by DOX can be related to the drug inhibiting effect on the proliferation of colon and SI organoids.

Tissue-specific DEGs resulting from the STEM analysis in the most significant time-dependent clusters were further evaluated for the two higher concentrations (Table 3). The novel DEGs found in the colon included *DHRS2*, *RGCC*, *LAMP3*, *TP53I3*, *TNFSF15*, *ABCA12* and *MFAP3L.* These genes are mainly involved in the regulation of cell cycle, gene expression, nuclear signalling pathways, cellular responses to oxidative stress, and metabolism of xenobiotics. The majority of these DEGs were upregulated in the colon organoids exposed to DOX. An exception was *TNFSF15*, which belongs to the TNF superfamily and whose role is linked to apoptosis modulation and signalling. SI novel specific DEGs included *CAPN8*, *CTNND1*, *MPRIP*, *TSPAN1*, *TPX2*, *MCM5*, *DHRS9*, *SLC2A3*, *PPP1R3C* and *MT1X*, which were all found to be downregulated in treated organoids. These genes are involved in several pathways from cell cycle and DNA replication to cell adhesion and signal transduction. Alterations on the expression levels of these tissue-specific genes can potentially be indicators of DOX-induced toxicity in the colon or SI cells. Further studies are required to establish how the DEGs can be used to detect DOX-induced toxicity in both colon and SI tissue of patients since these findings are new and there is no data available to support them. Additional pharmacogenomic studies should also be considered to investigate the impact of the genetic background on drug-gene effects [46] to validate the tissue-specific responses of colon and SI organoids as they derive from different donors. Nevertheless, it is still challenging to generate paired healthy colon and SI organoids as donors would need to undergo unnecessary surgical procedures, and these models are not commercially available.

Regarding the proteomics side, 19 in silico proteins were found associated with DOX-induced intestinal toxicity, the majority being involved in DOX metabolism and elimination, with few exceptions such as *ALDH1A1*, *TOP2A*, and *NOLC1*, as they are involved in cell growth, differentiation, and proliferation processes [47,48]. Comparison between proteomic and transcriptomic responses resulted in a summary of DOX-induced effects presented in Figure 6, starting from the entering of DOX into the cell to its elimination, through metabolism and generating the drug’s different metabolites, with consequent formation of radical oxygen species (ROS), and negative effects in several biological processes. It appears that genes encoding for enzymes involved in both metabolism and elimination of DOX and its metabolites were affected as they were found downregulated. Nevertheless, biological processes related to DOX metabolism into its different metabolites was not among the most significantly perturbed pathways, as these are not mechanisms that predominantly occur in the intestinal cells but rather in the liver. This could mean that DOX is less metabolized and eliminated from the organoids. As a result, the accumulation of this drug and its metabolites led to the formation of ROS, which caused mitochondria dysfunction, evidenced by the observed downregulation of complex I genes and the impairment of the respiratory electron transport and ATP synthesis pathway. Another consequence was the observed activation of oxidative stress-induced senescence pathway caused by not only the presence of ROS but also via p21 pathway upon upregulation of p53 and Cyclin D1, a mechanism also reported in a previous study [49]. Additionally, and due to oxidative stress, gene *TNIK*, an important activator of WNT target genes [50], was affected and led to perturbations in WNT signalling pathway, impairing cell growth and differentiation. DOX also contributed to upregulation of *DHSR2* followed by downregulation of *MDM4*, a known inhibitor of p53, thus in line with the observed p53 upregulation and activation of the p53 signalling pathway, as supported by previous studies [51,52]. Next, downregulation of *TPX2* led to increased levels of p53, as reported previously that depletion of *TPX2* is required during the synthesis of p53 [53]. In turn, upregulation of p53, apart from the already described effects on oxidative stress and cell cycle mechanisms, upregulates *RGCC*, a response associated with DNA damage that suppresses cell cycle progression [54]; inhibits *THBS1*, an inhibitor of angiogenesis processes [31]; and downregulates *MCM5*, involved in DNA replication. Similarly, impairment of signalling by retinoic acid, essential for cell growth and stem cell differentiation [55], was also observed after comparing the omics data since the DEGs involved, namely *DHSR9* and *ALDH1A1*, were found downregulated [47]. Moreover, membrane transporters of lipids and glucose were found to be perturbed as well. Gene encoding membrane protein *ABCA12*, responsible for the export of lipids, was upregulated, in line with the metabolism of lipids being affected by DOX in colon organoids. In turn, glucose transporter *SLC2A3* was found downregulated, which could mean that the cells are not taking in glucose necessary for glycolysis, in line with glycolysis being significantly affected in colon organoids exposed to DOX. Lastly, apoptosis and inflammation processes were not significantly affected pathways, despite the tendency of gene expression levels to increase. This shows that those pathways are not prevailing as mechanisms occurring in intestinal cells upon exposure to the drug. In summary, Figure 6 represents an overview of the potential mechanisms through which DOX exerts its toxicity in the intestinal cells.

Although clinical studies on DOX are lacking and the majority focus on cardiotoxicity, an attempt was made to compare the DEGs found in colon and SI-exposed organoids with the transcriptomic data available. Two of the DEGs mentioned above were found in cardiomyocytes exposed to DOX, namely *CCND1* (cyclin D1) and *TP53I3* (Tumour Protein P53 Inducible Protein 3). Cyclin D1 was found upregulated in cardiomyocytes of mice after a 16 h treatment with DOX [56]. Similarly, the expression levels of *CCND1* in the colon and SI cells increased over time and concentrations of DOX. In turn, the *TP53I3* gene was found in exposed cardiomyocytes that were originated from human embryonic stem cells [57]. These two genes, despite not being tissue specific, could be of more relevance in the investigation of gene responses to DOX effects as they seem to be implicated not only in cardiotoxicity but also in intestinal toxicity.

Overall, this study demonstrates the usefulness and potential of the intestinal organoid-based 3D culture model to provide new insights into the molecular mechanisms of DOX-induced toxicity in the intestinal tissue, as most studies focus on cardiotoxicity. DOX caused perturbations in cell cycle, oxidative stress, mitochondria function, activation of the p53 signalling pathway, signalling by retinoic acid and transport of molecules essential for energy metabolism, which in turn impaired the normal cell growth, proliferation and differentiation. Confirmation of the mode of action of DOX, as well as new findings on DOX-induced intestinal toxicity, are summarized in Figure 6. Promising new tissue-specific gene markers of DOX toxicity are also highlighted in Figure 6. Future studies should include the assessment of functional endpoints and transcriptomic responses at the intestinal level in cancer patients taking DOX monotherapy. This is important for the investigation of whether intestinal organoids can reflect these responses better than other cell or animal models, and to check for potential translatability to clinical settings. Pharmacogenomic studies, once the challenge of finding paired healthy colon and SI organoids is overcome, should also be considered to confirm the tissue-specific responses of colon and SI to DOX as patients can respond to therapies in different fashions due to the variability of genetic backgrounds. The elucidation of the underlying mechanisms of toxicity is a starting point for finding pharmacogenomics candidate markers. Furthermore, the new insights on DOX mechanisms of toxicity and gene responses in the intestinal cells are being applied for the development of predictive models of GI toxicity caused by drugs. Ultimately, and in the context of the transQST project, integration of this work with in silico tools will be useful for new drug design and to better assess the safety of drug candidates before clinical testing.

## 4. Materials and Methods

### 4.1. In Vitro Culture of Healthy Intestinal Organoids

Human healthy colon and small intestinal (SI) organoids were kindly provided by Boehringer Ingelheim Pharmaceuticals Inc. (Ridgefield, CT, USA) and established in a 3D culture at our laboratory. Colon and SI organoids were derived from the healthy tissue section of 67 and 74-year-old male donors, respectively, and purchased from Conversant Biologics (currently Discovery Life Sciences, Huntsville, Alabama, USA). The 3D in vitro culture of the colon and SI tissue was established following the methods described by Sato et al. [58]. Frozen organoids were recovered and cultured on a 24-well plate in complete crypt medium composed of advanced DMEM/F12 medium (Life Technologies, Bleiswijk, The Netherlands), Wnt3a conditioned medium, 1 µg/mL recombinant Human R-Spondin-1 (Peprotech, Hamburg, Germany), 10 mM nicotinamide (Merck, Darmstadt, Germany), 1× B27™ Supplement (50×) serum-free (Thermo Fisher Scientific, Waltham, MA, USA), 1.25 mM *N*-Acetyl-L-cysteine (Merck, Darmstadt, Germany), 50 ng/mL recombinant Human HB-EGF (Peprotech, Hamburg, Germany), 0.5 µM A 83–01 (Tocris, Abingdon, UK), 10 µM SB 202190 (p38i) (Merck, Darmstadt, Germany), 10 µM human [Leu^15^]-Gastrin I (Merck, Darmstadt, Germany), 1× Primocin (Thermo Fisher Scientific, Waltham, Massachusetts, USA), 0.1 µg/mL recombinant human Noggin (Peprotech, Hamburg, Germany) and 10 µM inhibitor Y-27632 (AbMole Bioscience, Houston, TX, USA) until the plate showed high confluency and signs of cell differentiation. At this stage, organoids were passaged, transferred, and cultured in 96-well plates.

Colon and SI organoids were passaged every 3–7 days, depending on the rate of growth and/or differentiation. Culture plates containing organoids were put on ice to promote easier disruption of the matrigel matrix (phenol-red free, Corning, NY, USA) and washed with cold basal culture medium composed of advanced DMEM/F12 medium, Glutamax 100×, HEPES buffer and FBS (Life Technologies, Bleiswijk, The Netherlands). Organoids were collected into 15 mL conical tubes and centrifuged at 300× *g* for 5 min, at 4 °C. The pellet was re-suspended in Tryple Express 1× (Life Technologies, Bleiswijk, The Netherlands) containing inhibitor Y-27632, followed by a quick vortex and two minutes incubation at 37 °C. Basal culture medium was then added to the tube and organoids were dissociated into single cells. Two more centrifugations at 800× *g* for 5 min, 4 °C, were performed to wash the pellet, which was re-suspended in ice-cold matrigel. Drops of 10–15 µL of matrigel containing organoids were seeded in pre-warmed culture plates and polymerized at 37 °C for 15–20 min. Pre-warmed complete crypt medium was added to each well and incubated at 37 °C, 5% CO_2_. The medium was refreshed every 2–3 days. Colon and SI organoids were also grown in Human IntestiCult™ Organoid Growth Medium (Stemcell, Cologne, Germany), prepared according to manufacturer’s instructions, to promote organoid differentiation. Differentiation was verified after 2–3 days, showing that organoids started to form buds that resembled intestine-like features, as described in our previous study [10].

### 4.2. Selection of DOX In Vitro Concentrations Based on PBPK Simulation

The Simcyp^®^ PBPK simulator (Version 18 Release 2, Certara UK Ltd., Sheffield, UK) was used to model and simulate DOX pharmacokinetics following intravenous (IV) dosing in humans. Based on the understanding of DOX physiochemical properties and absorption, distribution, metabolism, and excretion (ADME) in humans, the PBPK model was parameterized and verified using data from peer-reviewed literature. Details of the input parameters for the DOX PBPK model are listed in Table S1. The predictive performance of the DOX model was verified against DOX total plasma concentrations by simulating reported human dosing studies [21,22]. The studies used for performance verification were independent of data used to parameterise the model. To encompass a clinically relevant range of DOX exposure, 20 min IV infusions at doses of 2.5, 15, and 40 mg/m^2^ were simulated in a virtual population of North European Caucasian adults (*n* = 70, age 20 to 50 years, the proportion of females = 0.5). The minimum and maximum doses were selected based on 0.25-fold and 2-fold of the recommended weekly dosage of 10–20 mg/m^2^ [59].

The Simcyp in vitro data analysis toolkit (SIVA Version 3, Certara UK Ltd., Sheffield, UK) for virtual in vitro intracellular distribution (VIVD) [60] was used to predict in vitro distribution of DOX in human intestinal organoids. This informed a selection of nominal in vitro concentrations to achieve equivalent drug exposure between in vitro human intestinal organoid intracellular concentrations and in vivo gut concentrations after IV administration in humans, which is the common administration route in patients. Details of the input parameters for the DOX in vitro distribution model based on intestinal organoid study design and DOX physiochemical properties are in Table S2.

### 4.3. In Vitro Exposure to DOX

DOX was purchased from Merck (Darmstadt, Germany), with ≥99% purity. Intestinal organoids were seeded in 96-well plates in complete crypt medium for 2 days, after which it was replaced with Human IntestiCult Growth medium for 3 days to stimulate organoid differentiation, after which they were exposed to DOX. The selected concentrations were within the range of therapeutic doses except for the highest one due to previous organoid lack of response at lower concentrations of the drug. Differentiated intestinal organoids were exposed to 100 µL Human IntestiCult Growth medium with 1, 10, 30 and 60 µM DOX, selected based on Symcyp simulations, for 24, 48 and 72 h, and with no change of medium in between. Control wells were also included for all time points, including vehicle and untreated controls, consisting of organoids seeded in 100 µL IntestiCult medium with 0.1% DMSO and medium only, respectively. All exposures were performed in biological triplicates in 96-well plates. A blank reaction was added to the treatment layout, consisting of matrigel without organoids in 100 µL IntestiCult medium. Empty wells were filled with 200 µL PBS to avoid edge effects. After exposure, samples were collected to perform cytotoxicity assays and transcriptomic analyses.

### 4.4. Cytotoxicity Assays: ATP Measurement and Caspase 3/7 Activity

Measurement of the toxicity profile was performed using viability (ATP measurement) and apoptosis (caspase 3/7 activity) endpoints using 3D Celltiter-Glo and Caspase-Glo 3/7 (Promega, Madison, Wisconsin, USA), respectively, according to the manufacturer’s instructions. After each exposure time point, the medium was removed from the plates and replaced by 100 µL of each kit reagent to the appropriate wells, followed by homogenization of the matrigel. The plates were placed in a Scilogex MX-M 96 well plate shaker for 1 h (incubation time), at room temperature. Afterwards, samples were transferred to white opaque 96-well plates (Corning, NY, USA) and luminescence was measured in GloMax^®^ 96 Microplate Luminometer (Promega, Madison, WI, USA). Luminescence values corresponding to the levels of either ATP or caspase 3/7 activation, were transferred to GraphPad Prism 9.0 (GraphPad Software) and corrected for the blank reaction to eliminate possible interferences of the matrigel matrix in both curves. Statistical differences between conditions were calculated by applying the analysis of variance (ANOVA) test.

### 4.5. Image Analysis

In parallel to viability and caspase assays, image-based analyses of the human intestinal organoids’ morphology after treatment with DOX were performed in the 3D image analysis solution Ominer^®^ (Crown Bioscience Netherlands B.V.; Leiden, The Netherlands). These analyses aimed to support the cytotoxicity assessment data and to confirm changes in the size of the organoids and the percentage of cell death derived from the known mode of action of DOX. For this, organoids were grown and treated with the same conditions as described previously (see Section 4.3), i.e., the cells were grown in matrigel for a total of 5 days after which they were exposed to DOX for 1, 2 or 3 days. Untreated controls and vehicle controls were also included. After each time point, fixation and staining were performed to visualize the nuclei and actin cytoskeleton, by applying a solution containing Hoechst 33258, final concentration 0.4 µg/mL (Merck, Darmstadt, Germany) and Phalloidin-FITC, final concentration 0.1 µM (Merck, Darmstadt, Germany) [61]. Images were captured as z-stacks in an ImageXpress Micro XLS (Molecular Devices, Silicon Valley, CA, USA) wide-field microscope, using the 4x objective.

### 4.6. RNA Isolation from Intestinal Organoids

At each time point, the medium was removed from the plates and 200 µL of QIAzol Lysis reagent (Qiagen, Venlo, The Netherlands) was added into the wells to promote dissociation of the matrigel matrix and collection of each pellet to the respective tubes. This process was repeated several times, until all organoids were collected, ensuring a total volume of 700 µL of QIAzol Lysis reagent in each tube. Complete homogenization of organoids in the lysis reagent was reached by vigorous pipetting and vortex. RNA isolation was performed using the miRNeasy Mini Kit (Qiagen, Venlo, The Netherlands), following the manufacturer’s protocol for Animal Cells including a DNase treatment. Total RNA yield was measured on Nanodrop^®^ ND-1000 spectrophotometer (Thermo Fisher Scientific, Waltham, Massachusetts, USA) and RNA quality was confirmed using RNA Nanochips on a 2100 Bioanalyzer (Agilent Technologies, Leuven, Belgium). All samples with integrity number (RIN) >7 and total amount of RNA ≥200 ng were approved for RNA sequencing.

### 4.7. Library Preparation and mRNA Sequencing

Samples containing purified RNA were prepared for sequencing using the Lexogen SENSE mRNA library preparation kit (Lexogen, Vienna, Austria). After library preparation, the samples were sequenced on the NovaSeq 6000 system (Illumina, Eindhoven, The Netherlands). A pool of all DOX samples and controls was sequenced on the 2 lanes of an S1 flow cell. Untreated, vehicle controls and DOX samples were sequenced with an average of 10 to 15 million raw reads.

### 4.8. Pre-Processing and Data Analysis

For all samples, the first 12 bases of the 5′ end of all reads were removed using Trimmomatic version 0.33 [62], because the sequencing reads still contained Lexogen adapter sequences. Before and after trimming, the quality of the sequencing data was confirmed using FastQC version 0.11.3 [63] and only samples with satisfactory parameters were kept for downstream analysis. In addition, a cut-off of 5 million mapped reads was applied to all samples, as recommended for DGE analysis [29,30]. Following trimming, reads were aligned to the primary assembly of the human genome (Ensembl build v. 93 GRCh38) using Bowtie 1.1.1 and quantified with RSEM 1.3.1. The profile and behaviour of the samples were assessed according to the amount of (mapped) reads, hierarchical clustering, principal component analysis (PCA), and sample dispersion. Following quantification of the read counts, normalization was performed on the expected read counts from all samples and the contrast function from the R package DESeq 2 (v. 1.14.1) [64] was used to extract DEGs for each time point and concentration such that comparison between all conditions of the experiment is possible. For each specific time point, the following comparisons were performed: (a) untreated control vs. vehicle control; (b) 1 µM DOX vs. vehicle control; (c) 10 µM DOX vs. vehicle control; (d) 30 µM DOX vs. vehicle control; (e) 60 µM DOX vs. vehicle control. Bonferroni correction [65] was applied to the genes obtained, after which genes with adjusted *p* value <0.05 were considered as DEGs.

### 4.9. Proteome Analysis

Protein type targets of DOX were collected from open repositories such as ChEMBL [25], DrugBank [26], TTD [27], IUPHAR [28], PharmGKB [24] using a pre-constructed KNIME data science workflow [23]. The criteria for a target protein were individually adjusted to the guidelines of the corresponding database. For the list of target proteins, first-degree interactors were obtained from the MINT [66] and IntAct [67] protein-protein interaction databases, using the Reactome services [68]. A cut-off of 0.5 for the interacting scores secured that only reliable interactions with experimental evidence were taken into consideration. The curated list of targets and interacting proteins was filtered based on tissue-specific information. For that, the Proteomics DB was used. The output of the data mining is a table of target proteins and their first-degree interactors that can be connected to DOX and are expressed in the gut.

### 4.10. Pathway Analysis

The lists of DEGs obtained for each time point and concentration were used as input for pathway over-representation analysis (ORA) using ConsensusPathDB (CPDB) release 34 [69], considering a p value cut-off of 0.01. The Reactome database version 67 [70] and Kyoto Encyclopedia of Genes and Genomes (KEGG) [71] were selected as preferred databases for pathway analysis and interpretation of biological processes. Venn diagrams to compare genes in colon and SI organoids and gene plots of the most relevant DEGs were also generated using Venny 2.1 [72] (https://bioinfogp.cnb.csic.es/tools/venny/ (accessed on 27 December 2021)) and Excel, respectively. Moreover, the tool Short Time Series Expression Miner (STEM) [73] was used to classify the DEGs according to their differential expression degrees during the exposure to DOX, considering a *p* value ≤0.05. This analysis aimed at identifying the time-dependency of the transcriptome response, following the hypothesis that over time, gene responses are functionally interrelated. The list of genes obtained from the most significant clusters in the STEM tool was further analysed by performing a List Enrichment Network using the NetworkAnalyst 3.0 [74]. Ultimately, the most relevant DEGs and proteins obtained from transcriptomics and proteomics data, respectively, were compared and visually summarised using BioRender illustration tool [75] (BioRender.com (accessed on 23 April 2021)).

## Figures and Tables

**Table 1:** Nominal in vitro DOX concentrations that reach intracellular concentrations equivalent to physiologically based pharmacokinetic (PBPK) predicted human in vivo gut tissue maximum concentration (C_max_) after various intravenous (IV) doses.

IV Dose Infused Over 20 min in Human (mg/m^2^)	In Vivo Gut Tissue Total C_max_ (µM)	In Vitro Nominal Concentration (µM)
2.50	1.06	0.96
15.00	6.36	5.76
40.00	17.00	15.40

**Table 2:** Overview of the most relevant pathways and respective q values. Pathways identified by ORA in CPDB, considering Reactome and KEGG databases, and organized into main groups of biological pathways. Alterations in these pathways can be observed over time and concentration of DOX. The q values were obtained after using the false discovery rate method and they were considered as significant when below 0.05 (values in bold) or not applicable (NA) as the respective pathways were not present for a certain condition after CPDB analysis.

Name of the Pathway	Pathway Source	Time of Exposure (h)	DOX Conc. (µM)	*q* Value	
				Colon	*SI*
**Cell Cycle**	Reactome	24	1	**2.74 × 10^−20^**	NA
			10	**1.64 × 10^–13^**	**3.04 × 10^–4^**
			30	**2.57 × 10^–8^**	0.14
			60	**2.04 × 10^–6^**	NA
		48	1	NA	NA
			10	**4.19 × 10^–3^**	**9.96 × 10^–3^**
			30	**1.08 × 10^–3^**	0.16
			60	NA	0.06
		72	1	NA	NA
			10	**0.01**	0.06
			30	**0.04**	0.24
			60	0.09	NA
**Cell cycle—DNA repair**	Reactome	24	1	**0.027**	NA
			10	**2.81 × 10^–8^**	**2.16 × 10^–3^**
			30	**1.04 × 10^–3^**	NA
			60	**5.19 × 10^–3^**	NA
		48	1	NA	NA
			10	**0.04**	**1.08 × 10^–3^**
			30	**0.01**	NA
			60	0.06	NA
		72	1	NA	NA
			10	NA	NA
			30	NA	NA
			60	0.25	NA
**Gene expression—the p53 signalling**	KEGG	24	1	**1.32 × 10^–5^**	NA
			10	**7.84 × 10^–13^**	**0.02**
			30	**2.79 × 10^–7^**	**7.77 × 10^–3^**
			60	**4.99 × 10^–5^**	0.11
		48	1	**0.03**	NA
			10	**6.60 × 10^–5^**	0.05
			30	**3.92 × 10^–5^**	0.12
			60	**8.63 × 10^–4^**	NA
		72	1	0.06	NA
			10	**2.65 × 10^–6^**	0.17
			30	**8.25 × 10^–7^**	NA
			60	**4.56 × 10^–6^**	NA
**Epigenetic regulation of gene expression—DNA methylation**	Reactome	24	1	NA	**0.04**
			10	**6.63 x 10–^14^**	**4.95 × 10^–11^**
			30	**3.98 × 10^–14^**	**8.08 × 10^–6^**
			60	**4.44 × 10^–11^**	**1.03 × 10^–3^**
		48	1	NA	NA
			10	**2.60 × 10^–7^**	**2.58 × 10^–6^**
			30	**3.12 × 10^–8^**	**7.41 × 10^–5^**
			60	**5.58 × 10^–7^**	**7.49 × 10^–4^**
		72	1	NA	NA
			10	**0.02**	NA
			30	**2.96 × 10^–3^**	NA
			60	**9.08 × 10^–5^**	NA
**Metabolism of carbohydrates—Glycolysis/Gluconeogenesis**	KEGG	24	1	NA	NA
			10	NA	NA
			30	NA	0.15
			60	NA	NA
		48	1	NA	NA
			10	**6.81 × 10^–3^**	NA
			30	**0.02**	0.11
			60	**0.04**	0.14
		72	1	NA	NA
			10	**6.43 × 10^–4^**	NA
			30	**6.84 × 10^–5^**	NA
			60	**7.95 × 10^–6^**	NA
**Metabolism—Respiratory electron transport, ATP synthesis by chemiosmotic coupling, and eat production by uncoupling proteins**	Reactome	24	1	NA	NA
			10	NA	NA
			30	NA	NA
			60	**6.24 × 10^–3^**	NA
		48	1	NA	NA
			10	NA	NA
			30	NA	NA
			60	0.05	NA
		72	1	NA	NA
			10	NA	NA
			30	NA	NA
			60	0.20	NA
**Metabolism of lipids**	Reactome	24	1	NA	NA
			10	0.15	0.09
			30	**7.01 × 10^–3^**	0.15
			60	0.07	NA
		48	1	NA	NA
			10	**8.49 × 10^–5^**	NA
			30	**5.41 × 10^–5^**	NA
			60	**9.11 × 10^–6^**	NA
		72	1	NA	NA
			10	**1.00 × 10^–3^**	NA
			30	**5.19 × 10^–4^**	NA
			60	**3.91 × 10^–3^**	NA
**Metabolism of amino acids and derivatives**	Reactome	24	1	NA	NA
			10	0.08	**0.03**
			30	0.15	**4.46 × 10^–17^**
			60	0.07	**1.29 × 10^–36^**
		48	1	0.13	NA
			10	**1.00 × 10^–3^**	NA
			30	0.05	**6.77 × 10^–27^**
			60	NA	**1.99× 10^–4^**
		72	1	NA	NA
			10	**3.12 × 10^–4^**	NA
			30	**2.13 × 10^–10^**	NA
			60	**6.34 × 10^–7^**	NA
**Cellular responses to external stimuli—oxidative stress induced senescence**	Reactome	24	1	NA	0.05
			10	**2.12 × 10^–9^**	**9.19 × 10^–9^**
			30	**2.78 × 10^–11^**	**3.28 × 10^–6^**
			60	**8.00 × 10^–10^**	**9.02 × 10^–5^**
		48	1	NA	NA
			10	**1.45 × 10^–5^**	**1.88 × 10^–5^**
			30	**3.09 × 10^–7^**	**1.82 × 10^–4^**
			60	**4.63 × 10^–6^**	**2.00 × 10^–3^**
		72	1	NA	NA
			10	0.05	NA
			30	**4.82 × 10^–3^**	NA
			60	**6.23 × 10^–4^**	NA

**Table 3:** The most significantly altered DEGs selected after analysis with STEM, considering cluster 1 and similar, and the exposure to 30 and 60 µM DOX concentrations over time are described. The DEGs are either specific to colon or SI. The complete name of the DEGs as well as the main pathways in which they are involved.

Concentration (µM)	Gene Symbol	Name	Direction of Expression (Control vs. DOX)	Main Pathway(s) Involved
Colon				
**30**	*DHRS2*	Dehydrogenase/reductase SDR family member 2	↑	Metabolism of several compounds
	*RGCC*	Regulator of cell cycle	↑	Regulation of cell cycle progression via p53
	*LAMP3*	Lysosome-associated membrane glycoprotein 3	↑	Gene expression; adaptive immunity
	*TP53I3*	Tumour Protein P53 Inducible Protein 3	↑	Cellular responses to oxidative stress
	*TNFSF15*	TNF Superfamily Member 15	↑	Apoptosis modulation and signalling
**60**	*ABCA12*	ATP Binding Cassette Subfamily A Member 12	↑	Transport of molecules
	*RGCC*	Regulator of cell cycle	↑	Regulation of cell cycle progression via p53
	*DHRS2*	Dehydrogenase/reductase SDR family member 2	↑	Metabolism of several compounds
	*MFAP3L*	Microfibril Associated Protein 3 Like	↑	Nuclear signalling pathways (EGFR and MAPK)
	*LAMP3*	Lysosome-associated membrane glycoprotein 3	↑	Gene expression; adaptive immunity
SI				
**30**	*CAPN8*	Calpain 8	↓	Degradation of the extracellular matrix
	*CTNND1*	Catenin Delta 1	↓	Cell adhesion and signal transduction
	*MPRIP*	Myosin Phosphatase Rho Interacting Protein	↓	Signalling by BRAF and RAF fusions
	*TSPAN1*	Tetraspanin 1	↓	Regulation of cell development, activation, growth and motility
	*TPX2*	Microtubule Nucleation Factor	↓	Cell cycle and Regulation of p53 activity
**60**	*MCM5*	Minichromosome Maintenance Complex Component 5	↓	DNA replication
	*DHRS9*	Dehydrogenase/Reductase 9	↓	Metabolism
	*SLC2A3*	Solute Carrier Family 2 Member 3	↓	Transport of glucose
	*PPP1R3C*	Protein Phosphatase 1 Regulatory Subunit 3C	↓	Glycogen synthesis
	*MT1X*	Metallothionein 1X	↓	Metallothioneins bind metals

Legend: ↑ - upregulated; ↓ - downregulated.

**Table 4:** Tissue-specific target proteins of DOX.

UniProt Accession	Gene Name	Protein Name
O43488	AKR7A2	Aflatoxin B1 aldehyde reductase member 2
O75251	NDUFS7	NADH dehydrogenase [ubiquinone] iron–sulphur protein 7, mitochondrial
O75306	NDUFS2	NADH dehydrogenase [ubiquinone] iron–sulphur protein 2, mitochondrial
O75489	NDUFS3	NADH dehydrogenase [ubiquinone] iron–sulphur protein 3, mitochondrial
O75828	CBR3	Carbonyl reductase [NADPH] 3
P00352	ALDH1A1	Retinal dehydrogenase 1
P02768	ALB	Albumin
P11388	TOP2A	DNA topoisomerase 2-alpha
P14550	AKR1A1	Aldo-keto reductase family 1 member A1
P15559	NQO1	NAD(P)H dehydrogenase [quinone] 1
P16083	NQO2	Ribosyldihydronicotinamide dehydrogenase [quinone]
P16152	CBR1	Carbonyl reductase [NADPH] 1
P16435	POR	NADPH--cytochrome P450 reductase
P29474	NOS3	Nitric oxide synthase, endothelial
P29475	NOS1	Nitric oxide synthase, brain
Q14978	NOLC1	Nucleolar and coiled-body phosphoprotein 1
Q15311	RALBP1	RalA-binding protein 1
Q92887	ABCC2	Canalicular multispecific organic anion transporter 1
Q9NUT2	ABCB8	Mitochondrial potassium channel ATP-binding subunit

## Data Availability

The cytotoxicity data generated and analysed during the current study are available in the BioStudies repository (www.ebi.ac.uk/biostudies/studies/S-TQST114 (accessed on 27 December 2021)). The transcriptomic data generated and analysed during the current study will be publicly available on ArrayExpress repository (www.ebi.ac.uk/arrayexpress/ (accessed on 27 December 2021)) with accession number E-MTAB-11297.

## Supplementary Materials

The following supporting information can be downloaded at: https://www.mdpi.com/article/10.3390/ijms23031286/s1. References [22,35,60,76,77,78,79,80,81] are cited in the Supplementary Materials.
