# Supplementary material for: Unravelling Mechanisms of Doxorubicin-Induced Toxicity in 3D Human Intestinal Organoids
Source: Int J Mol Sci. 2022 Jan 24;23(3):1286. doi: 10.3390/ijms23031286 (PMC8836276; doi:10.3390/ijms23031286)
Supplement: Supplementary file 1 [file ijms-23-01286-s001.zip › ijms-1553393-supplementary.pdf]

Supplementary Materials

Title: Unravelling mechanisms of doxorubicin-induced toxicity in 3D human intestinal organoids

Journal: International Journal of Molecular Sciences

Authors: Daniela Rodrigues*, Luke Coyle, Barbara Füzi, Sofia Ferreira, Heeseung Jo, Bram Herpers, Seung-Wook Chung, Ciarán Fisher, Jos C.S. Kleinjans, Danyel Jennen, Theo M. de Kok

*corresponding author: Department of Toxicogenomics, GROW School for Oncology and Developmental Biology, Maastricht University, Maastricht, the Netherlands. d.rodrigues@maastrichtuniversity.nl, ORCID ID: 0000-0001-5527-6627

Table S1. Input parameters for doxorubicin human physiologically based pharmacokinetic (PBPK) modelling in Simcyp simulator.

	Parameters and models	Doxorubicin	Source
**Physiochemical properties**	Molecular weight (g/mol)	543.53	Hanke et al 2018 [22]
	Log P_{ow}	1.27	Hanke et al 2018 [22]
	Compound type	Monoprotic Base	
	pK_a	8.2	Hanke et al 2018 [22]
	Blood-to-plasma ratio	0.76	Simcyp predicted
	Fraction unbound in plasma	0.28	Lombardo et al 2018 [76]
**Absorption**	Absorption model	Advanced Dissolution, Absorption and Metabolism (ADAM) model	
	Fraction unbound in enterocyte	1	Assumed maximum unbound compound in enterocyte
	Caco-2 6.5 : 7.4 passive & active permeability (10^{-6} cm/s)	0.16	Yee 1997 [77]
	Caco-2 6.5 : 7.4 passive & active permeability; Cimetidine as reference (10^{-6} cm/s)	3.06	Yee 1997 [77]
	Caco-2 6.5 : 7.4 passive & active permeability; Propranolol as reference (10^{-6} cm/s)	27.5	Yee 1997 [77]
	Human jejunum effective permeability (10^{-4} cm/s)	0.1223	Simcyp predicted
**Distribution**	Distribution model	Full PBPK	
	Volume of distribution at steady state (L/kg)	22.858	Lombardo et al 2018 [76]
	Prediction method	Rodgers & Rowland model [78, 79]	
	Kp Scalar	18	Scalar adjusted to reach observed V_{ss} from Lombardo et al 2018 [76]
**Elimination**	Clearance type	Enzyme kinetics	
	Hepatocyte *in vitro* intrinsic clearance (μL/min/10^6)	10	Adjusted to match observed clearance [35, 80]
	Biliary *in vitro* intrinsic clearance (μL/min/10^6)	20	Adjusted based on fraction excreted to faeces from Hanke 2018 [22]

Renal clearance (L/h)	3	Adjusted based on fraction excreted to urine from Hanke 2018 [22]
Additional systemic clearance	24	Adjusted based on overall excretion from Lombardo et al 2018 [76]

Table S2. Doxorubicin virtual *in vitro* distribution (VIVD) [60] model input parameters in Simcyp's *in vitro* data analysis (SIVA) toolkit based on human gut cell composition and human intestinal organoid culture condition.

	Parameters and models	Doxorubicin	Source
**Physiochemical properties**	Molecular weight (g/mol)	543.53	Hanke et al 2018 [22]
	Log P_{ow}	1.27	Hanke et al 2018 [22]
	Compound type	Monoprotic Base	
	pK_a	8.2	Hanke et al 2018 [22]
	Blood-to-plasma ratio	0.76	Simcyp predicted
	Fraction unbound in the culture medium	1	Foetal bovine serum not used in experiment
	Henry's Law Constant at 25 °C ($Pa.m^3/mol$)	2.22915×10^{-18}	EPI Suite V4.1.1 predicted [81]
	Aqueous solubility (mg/L)	10000	ChemSpider *
**Media parameters**	Culture media pH	7.4	Default
	Fraction of foetal bovine serum	1	Foetal bovine serum not used in experiment
	Foetal bovine serum pH	7	Default
	Albumin mass in 1L of foetal bovine serum (g)	0	Foetal bovine serum not used in experiment
	Concentration of triacylglyceride in foetal bovine serum (mmol/L)	0	Foetal bovine serum not used in experiment
	Volume of culture media per well (μL)	100	Experiment
**Culture parameters**	Diameter of culture vessel well (mm)	6.4	Experiment
	Total volume of culture vessel well (μL)	360	Experiment
	Culture temperature (°C)	37	Experiment
	Cell diameter (μm)	5.3192	Experiment **
	Cell number	3000	Experiment

* http://www.chemspider.com/Chemical-Structure.29400.html

** calculated based on average organoid area (4000 μm^2) and number of cells per organoid (180 cells)

Table S3. Identified DEGs whose expression was significantly affected after exposure to DOX and respective up- (↑) or downregulation (↓) in colon and in SI organoids.

Gene Symbol	*Pathways involved/Function*	*Trend of expression control vs treated in colon and SI*
**In both organoid types**		
ABCC2	Transport of molecules	↓/↓
CCND1	Cell cycle/proliferation	↑/↑
H2BC11	Cell cycle	↓/↓
H4C8	DNA methylation	↑/↑
MDM4	p53 signalling pathway	↓/↓
P53	p53 signalling pathway; cell cycle	↑/↑
THBS1	p53 signalling pathway	↑/↑
TNIK	Oxidative stress-induced senescence	↓/↓
**In colon organoids only**		
ABCA12	Transport of molecules (lipids)	↑
ALDH1A1	Signalling by retinoic acid; cell growth and differentiation	↓
CBR1	DOX metabolism	↓
DHSR2	Metabolism of several compounds	↑
NDUFS2	Electron transport chain	↓
NQO1	DOX metabolism	↓
NQO2	DOX metabolism	↓
RGCC	Regulation of cell cycle	↑
TNFSF15	Apoptosis modulation and signalling	↑
**In SI organoids only**		
DHSR9	Metabolism of several compounds	↓
SLC2A3	Transport of glucose	↓
TPX2	Cell cycle; regulation of p53 activity	↓
TSPAN1	Cell growth, differentiation and proliferation	↓

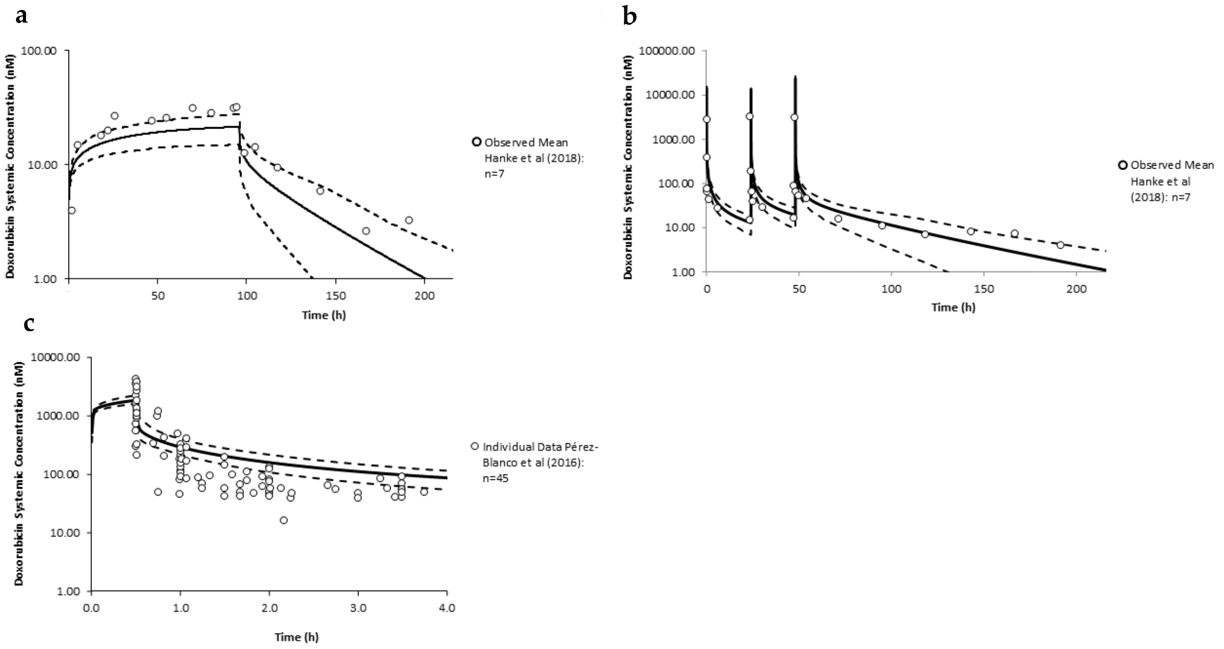

Figure S1. Verification of doxorubicin human PBPK model: predicted mean (black solid line) and observed (circle) doxorubicin plasma concentration following doxorubicin doses of a) single 36mg/m² intravenous (IV) infusion over 96 hours; b) three 30mg/m² IV bolus doses 24 hours apart; c) single 30mg/m² IV infusion over 0.5 hours. The dashed lines refer to the predicted 5th and 95th percentiles.

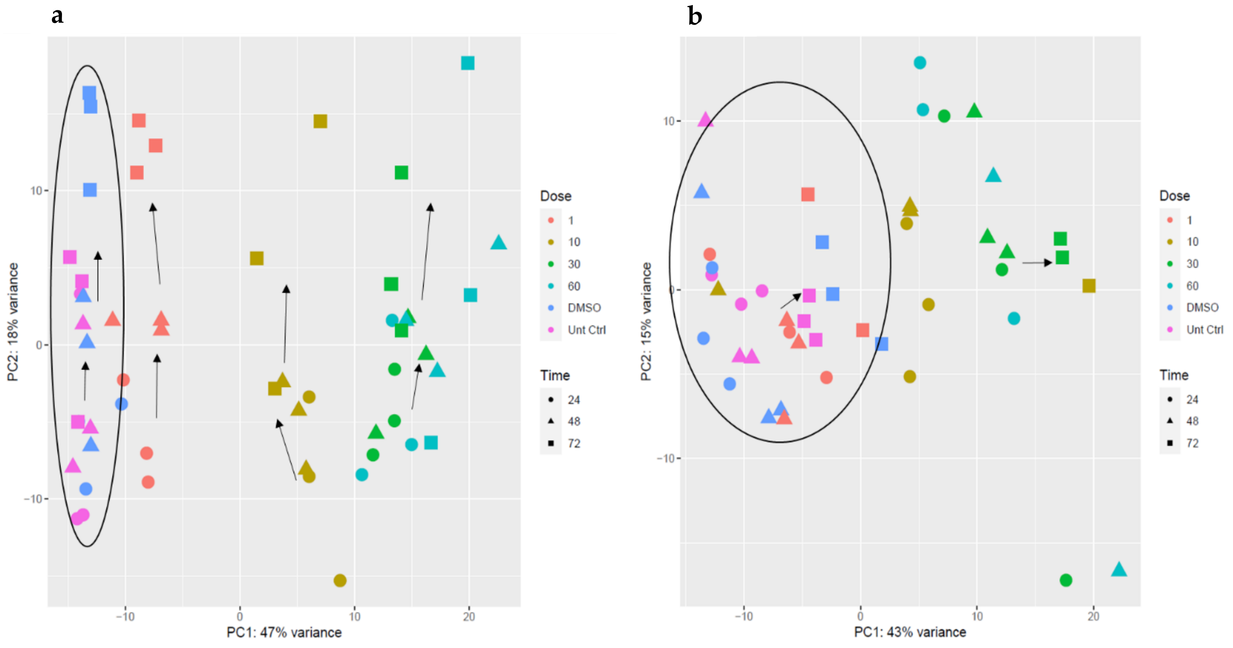

Figure S2. PCA score scatter plot obtained for the mRNA transcriptomic analysis of samples collected from colon organoids (a) and SI organoids (b). The direction of the arrows indicates the evolution in time of the samples (24h → 48h → 72h). In colon PCA, the cluster on the left comprises non-treated samples (untreated and vehicle controls at all time points); in SI PCA, the cluster on the left comprises non-treated samples and 1µM DOX. Legend: untreated controls are in pink; vehicle controls are in dark blue; 1µM DOX is in red; 10µM DOX is in yellow; 30µM DOX is in green; 60µM DOX is in light blue.

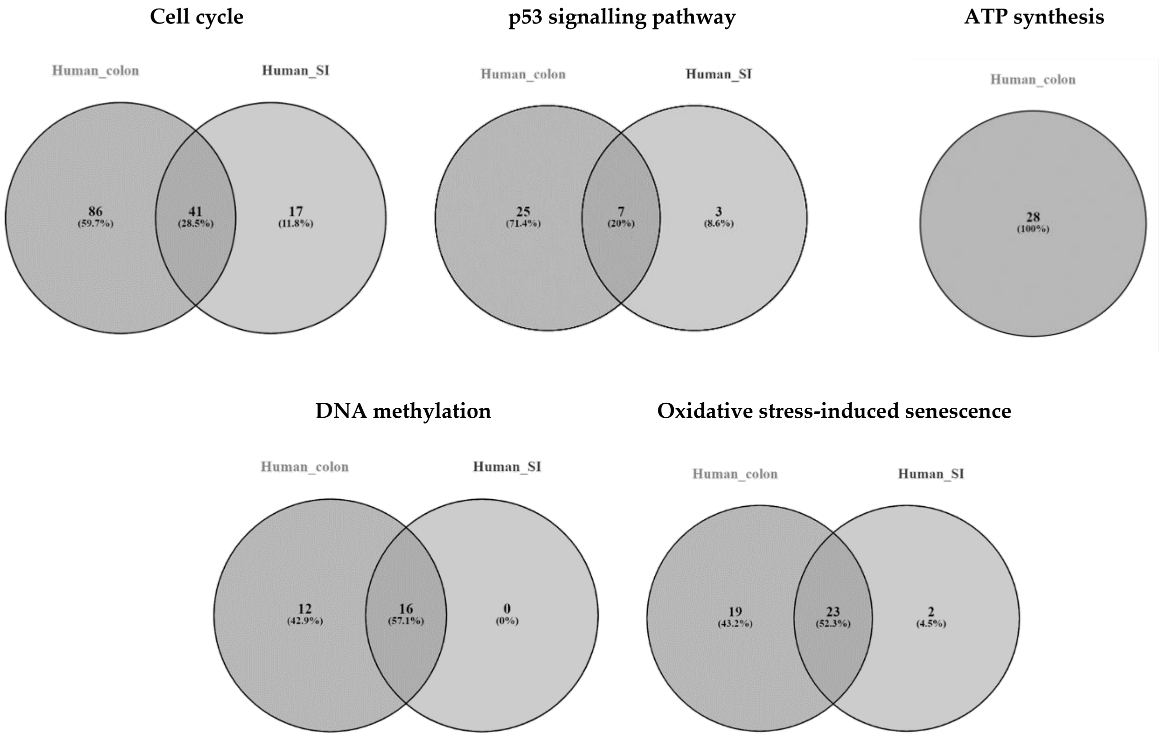

Figure S3. Venn diagrams comparing all the DEGs affected at all time points and DOX concentrations, between colon and SI organoids. DEGs are involved in cell cycle, p53 signalling pathway, ATP synthesis, DNA methylation and oxidative stress-induced senescence.

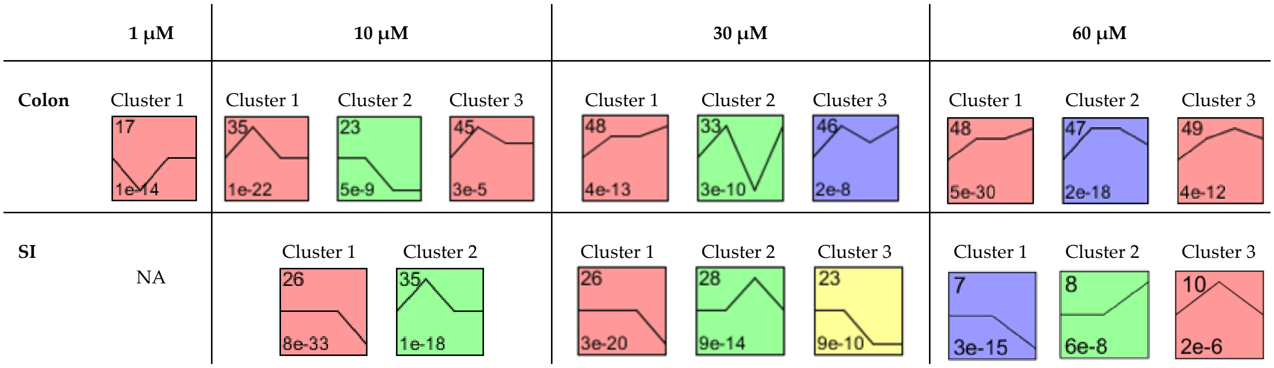

Figure S4. Correlated expression clusters were discovered using the short time-series expression miner (STEM) algorithm for time-series profile matching. Profiles were ordered based on the p-value significance of the number of genes versus expected. The clusters were selected for each concentration of DOX in both organs. NA, not available.

References

22. Hanke, N., et al., *A physiologically based pharmacokinetic (PBPK) parent-metabolite model of the chemotherapeutic zoptarelin doxorubicin-integration of in vitro results, Phase I and Phase II data and model application for drug-drug interaction potential analysis.* Cancer Chemother Pharmacol, 2018. **81**(2): p. 291-304.
35. Mross, K., et al., *Pharmacokinetics and metabolism of epidoxorubicin and doxorubicin in humans.* J Clin Oncol, 1988. **6**(3): p. 517-26.

60. Fisher, C., et al., *VIVD: Virtual in vitro distribution model for the mechanistic prediction of intracellular concentrations of chemicals in in vitro toxicity assays.* Toxicol In Vitro, 2019. **58**: p. 42-50.
76. Lombardo, F., G. Berellini, and R.S. Obach, *Trend Analysis of a Database of Intravenous Pharmacokinetic Parameters in Humans for 1352 Drug Compounds.* Drug Metab Dispos, 2018. **46**(11): p. 1466-1477.
77. Yee, S., *In vitro permeability across Caco-2 cells (colonic) can predict in vivo (small intestinal) absorption in man--fact or myth.* Pharm Res, 1997. **14**(6): p. 763-6.
78. Rodgers, T., D. Leahy, and M. Rowland, *Physiologically based pharmacokinetic modeling 1: predicting the tissue distribution of moderate-to-strong bases.* J Pharm Sci, 2005. **94**(6): p. 1259-76.
79. Rodgers, T. and M. Rowland, *Physiologically based pharmacokinetic modelling 2: predicting the tissue distribution of acids, very weak bases, neutrals and zwitterions.* J Pharm Sci, 2006. **95**(6): p. 1238-57.
80. Speth, P.A., Q.G. van Hoesel, and C. Haanen, *Clinical pharmacokinetics of doxorubicin.* Clin Pharmacokinet, 1988. **15**(1): p. 15-31.
81. Meylan WM, H.P. *HENRYWIN 3.10*. 2000.
